# MicroRNA expression and its implications for diagnosis and therapy of tongue squamous cell carcinoma

**DOI:** 10.1111/jcmm.12650

**Published:** 2015-10-24

**Authors:** Xin Yu, Zheng Li

**Affiliations:** ^1^ Department of Orthopaedic SurgeryPeking Union Medical College HospitalChinese Academy of Medical Sciences and Peking Union Medical CollegeBeijingChina

**Keywords:** tongue squamous cell carcinoma, microRNA, miRNA, oncogene, tumour suppressor gene

## Abstract

Tongue squamous cell carcinoma (TSCC) is the most common type of oral squamous cell carcinomas and is well known for its high rate of lymph nodal metastasis. Despite the identification of many molecular mechanisms in TSCC, the number of deaths associated with TSCC increased during the past 5 years. MicroRNAs (miRNAs) are a family of small non‐coding RNA molecules, which regulate gene expression by either translational inhibition or mRNA degradation. miRNAs have been proven to be key regulators of various biological and pathological processes including cell proliferation, development and tumourigenesis. Increasing evidence has demonstrated that the deregulated miRNAs are implicated in the diagnosis and treatment of TSCC. In this review, we summarized the expressions and roles of miRNAs in TSCC and comment on the potential roles of miRNAs in diagnosis, prognosis and treatment of this malignancy.

## Introduction

Oral cancer represents the sixth most frequent solid cancer around the world, with estimated ~300,000 new cases and 130,000 deaths annually worldwide. Tongue squamous cell carcinoma (TSCC) is the most common type of oral cancers 1, 2, 3, 4. Tongue squamous cell carcinoma frequently leads to the malfunction of mastication, speech and deglutition. Lymph node and distant metastasis are the most reliable adverse prognostic factors in TSCC patients 5, 6, 7, 8. Despite recent advances in its diagnostic techniques and therapeutic managements, the number of deaths associated with TSCC increased by over 10% during the past 5 years 9, 10, 11, 12. It is, therefore, of paramount importance to understand the molecular pathways of carcinogenesis or progression to develop novel therapeutic strategies of TSCC.

MicroRNAs (miRNAs), approximately 18 and 22 nucleotides (nt) in length, are endogenous, highly conserved, non‐coding RNA molecules. MicroRNAs negatively regulate gene expression by repressing translation or by direct sequence‐specific cleavage through the action of the RNA‐induced silencing complex following binding to the 3′‐untranslated region of mRNA 13, 14, 15, 16, 17, 18. Accumulating studies have demonstrated miRNAs play crucial roles in many biological and pathological processes, including development, cell proliferation, apoptosis, invasion, metabolism, response to stress and morphogenesis 19, 20, 21, 22, 23. Increasing evidence suggests that miRNAs are frequently deregulated in various cancers such as gastric cancer, osteosarcoma, breast cancer, glioblastoma, hepatocellular carcinoma, Ewing's sarcoma and renal cell carcinoma 24, 25, 26, 27, 28, 29, 30, 31. In this review, we focus on recent results related to miRNAs in TSCC development and discuss the potential use of miRNAs as prognostic biomarkers or treatment strategies for TSCC.

## miRNA expression profiling in tongue squamous cell carcinoma

The first report on miRNA expression profiling in TSCC was performed in tissues of TSCC and the adjacent normal epithelia tissues using Taqman‐based miRNA assays. The expression patterns of 156 mature miRNAs in TSCC were investigated. miR‐133a and miR‐133b were significantly down‐regulated in TSCC cells in comparison with the paired normal epithelial cells 32. Another study analysed the miRNA expression in 10 cases of TSCC using microarray 1. A marked difference in expression was observed in 71 miRNAs between TSCCs and normal tongue tissues. Using 2‐fold expression difference as a cut‐off level, they identified 10 up‐regulated miRNAs and 15 down‐regulated ones in four cases of early TSCC, whereas 26 up‐regulated miRNAs and 2 down‐regulated ones in the other six cases of advanced tumours. Liu *et al*. also identified a panel of differentially expressed miRNAs in paired OTSCC cell lines with different metastatic potential using miRNA expression microarray 33. It is well known that resistance to anticancer agents usually leads to failure of chemotherapy. Another study performed miRNA microarray to compare the differential miRNAs levels between the cisplatin‐sensitive TSCC line (Tca8113) and its cisplatin‐resistant subline (Tca/cisplatin) 34. Nineteen out of the 480 miRNAs were found differentially expressed between the Tca8113 and Tca/cisplatin cells. The expression of 836 miRNAs was analysed from 21 TSCC patitents and 8 controls using the miRNA microarray for 35. With the use of a linear regression model they identified 54 differentially expressed miRNAs in tongue cancer. Furthermore, Sun *et al*. 36. established stable chemotherapy‐resistant TSCC cell lines CAL27‐res and SCC25‐res by exposing the parental CAL27 and SCC25 lines to escalating concentrations of cisplatin for 6 months. Then, miRNA microarray was performed between them. A marked difference in expression was observed in 26 miRNAs between CAL27‐res cells and the parent line, including 14 up‐regulated and 12 down‐regulated miRNAs (Table 1).

**Table 1:** miRNA expression profiles in TSCC

Num	Method	Sample	Up‐regulated	Down‐regulated	References
1	Microarray	Primary TSCC		miR‐133a, miR‐133b	32
2	Microarray	Primary TSCC	let‐7a, miR‐16, miR‐203, miR‐21, miR‐27a, miR‐27b, miR‐31, miR‐17‐5p, miR‐195, miR‐23a	miR‐103, miR‐189, miR‐61, miR‐148a, miR‐184, miR‐26b, miR‐320, miR‐451, miR‐494, miR‐513, miR‐519b, miR‐525, miR‐202, miR‐342	1
3	Microarray	Cell lines (UM1/UM2)	miR‐18b, miR‐19a, miR‐20b, miR‐31, miR‐106a, miR‐148b, miR‐197, miR‐203, miR‐205, miR‐342, miR‐370, miR‐532, miR‐560, miR‐564, miR‐564‐pre, miR‐566, miR‐574, miR‐595, miR‐619‐pre, miR‐638, miR‐638‐pre, miR‐662‐pre	miR‐7, miR‐30a‐5p, miR‐30e‐3p, miR‐34b, miR‐34c, miR‐96, miR‐99a, miR‐99b, miR‐100, miR‐125a, miR‐125b, miR‐130a, miR‐130b, miR‐138, miR‐155, miR‐181c, miR‐181d, miR‐221, miR‐222, miR‐224, miR‐503, miR‐565‐pre, miR‐594‐pre	33
4	Microarray	(Tca/cisplatin cells)/Tca8113	Let‐7c, let‐7d, let‐7e, let‐7g, miR‐20b, miR‐23a, miR‐30d, miR‐181d, miR‐188, miR‐214, miR‐373, miR‐432, miR‐498, miR‐518c, miR‐584, miR‐608, miR‐628	miR‐21, miR‐342	34
5	Microarray	CAL27‐res/CAL27	let‐7d, miR‐122, miR‐193a‐5p, miR‐30c‐1, miR‐342‐3p, miR‐342‐5p, miR‐452, miR‐486‐3p, miR‐518b, miR‐628‐3p, miR‐663, miR‐675, miR‐877	let‐7b, let‐7e, let‐7f, let‐7i, miR‐15b, miR‐200b, miR‐20a, miR‐21, miR‐27b, miR‐886‐3p, miR‐93, miR‐98	36

UM1, UM2 (TSCC cell lines): aggressive than UM2 in term of cell invasion; Tca8113: cisplatin‐sensitive tongue squamous cell carcinoma line; Tca/cisplatin: its cisplatin‐resistant subline; CAL27: TSCC cell line; CAL27‐res: chemotherapy‐resistant TSCC cell line; TSCC: tongue squamous cell carcinoma.

## Up‐regulated miRNAs in tongue squamous cell carcinoma

Potential oncogenic functions of miR‐184 were evaluated in TSCC. Overexpression of miR‐184 was validated in 20‐paired tongue SCC and normal tissues. Plasma miR‐184 levels were also significantly higher in TSCC patients in comparison with normal individuals, and the levels were significantly reduced after surgical removal of the primary tumours. Inhibition of miR‐184 in tongue SCC cell lines could reduce cell proliferation rate and induce apoptosis. Down‐regulation of c‐Myc was observed in two cell lines in response to miR‐184 inhibitor 37. miR‐21 is an independent prognostic indicator for TSCC, and may play a role in TSCC development by inhibiting cancer cell apoptosis partly *via* tropomyosin 1 (TPM1) silencing. miR‐21 is overexpressed in TSCC relative to adjacent normal tissues. The level of miR‐21 is reversely correlated with TPM1 and PTEN (phosphatase and tensin homologue deleted on chromosome ten) expression and apoptosis of cancer cells. Multivariate analysis showed that miR‐21 expression is an independent prognostic factor indicating poor survival. Inhibiting miR‐21 with ASO in TSCC cell lines reduces survival and anchorage‐independent growth, and induces apoptosis in TSCC cell lines. Simultaneous silencing of TPM1 with siRNA only partially recapitulates the effect of miR‐21 ASO. Furthermore, repeated injection of miR‐21 ASO suppresses tumour formation in nude mice by reducing cell proliferation and inducing apoptosis 1. The expression of miR‐24 was up‐regulation of in TSCC. The miR‐24‐mediated change in Deadend1 expression suppressed the expression of cyclin‐dependent kinase inhibitor 1B, and also led to enhanced proliferation and reduced apoptosis in TSCC cells 38.

## Down‐regulated miRNAs in tongue squamous cell carcinoma

In contrast to the scarcity of reported up‐regulated miRNAs in TSCC, miRNA down‐regulation is more pervasive, suggestive of the tumour‐suppressing function of miRNAs as a whole. miR‐133a and miR‐133b were significantly reduced in TSCC cells in comparison with the paired normal epithelial cells. Overexpression of miR‐133a and miR‐133b inhibited the TSCC cell proliferation and enhanced the TSCC cell apoptotic. In addition, pyruvate kinase type M2 was identified as a direct target of miR‐133a and miR‐133b. Pyruvate kinase type M2 was overexpressed in tongue SCC and was associated with the down‐regulation of miR‐133a and miR‐133b 32. miR‐222 was down‐regualted in OTSCC and inhibits OTSCC cell invasion by regulating the matrix metalloproteinase 1 (MMP1) expression through both direct cis‐regulatory mechanism (targeting MMP1 mRNA) and indirect trans‐regulatory mechanism (indirect controlling of MMP1 gene expression by targeting superoxide dismutase 2) 33. miR‐7 suppressed tumourigenesis in TSCC through suppressing insulin‐like growth factor 1 receptor/Akt signalling pathway 39. miR‐138 played an important role in TSCC cell migration and invasion by concurrently targeting Rho‐related GTP‐binding protein C (RhoC) and ROCK2, and miR‐138 may serve as a novel therapeutic target for TSCC patients at risk of metastatic disease 40. Another study seeks to identify several targets of miR‐138 in TSCC, including chloride channel, nucleotide‐sensitive 1A (CLNS1A), G protein alpha inhibiting activity polypeptide 2, solute carrier family 20, member 1, eukaryotic translation initiation factor 4E binding protein 1 and RhoC. Therefore, a number of high‐confident miR‐138 target genes may play an important role in TSCC initiation and progression 41. miR‐99a was markedly decreased in OSCC tissues compared with the adjacent non‐tumour tissues. miR‐99a mimics significantly inhibited the proliferation of Tca‐8113 cells, a tongue squamous carcinoma cell line, and that miR‐99a mimics markedly induced the apoptosis of Tca‐8113 cells. Mammalian target of rapamycin was directly targeted by miR‐99a 3. Reduced miR‐195 expression was associated with tumour size and the clinical stage of TSCC tumours. Kaplan–Meier survival analysis indicated that the TSCC patients with reduced expression of miR‐195 had poor overall survival and in multivariable analyses low levels of miR‐195 emerged as an independent prognostic factor for this clinical outcome. Levels of miR‐195 expression were inversely correlated with the expression of Cyclin D1 and Bcl‐2. Overexpression of miR‐195 inhibited cell cycle progression, promoted apoptosis, and reduced Cyclin D1 and Bcl‐2 expression in two TSCC cell lines 42. Quantitative PCR showed that miR‐25‐3p expression in the TSCC cell lines and tissue specimen was significantly lower than that in the adjacent tissue. MTT and cell colony formation assays showed that after miR‐25‐3p overexpression, the proliferation of transfected Tca8113 was obviously attenuated. Western blot analysis and quantitative PCR showed that after miR‐25‐3p overexpression, p21(cip1) and p27(kip1) expressions were up‐regulated, while cyclinD1, AKT, FOXO1 expressions were down‐regulated, and AKT and FOXO1 phosphorylation was inactivated in the transfected Tca8113 cells 43. Jia *et al*. 44 demonstrated that miR‐26a and long noncoding RNA (lncRNA) MEG3 gene expression were both strongly reduced in TSCC compared with levels in matched non‐malignant tissues, and combined low expression levels of both miR‐26a and MEG3 emerged as an independent prognostic factor for poor clinical outcome in TSCC patients. Overexpression of miR‐26a or MEG3 in SCC‐15 and CAL27 cells inhibited cell proliferation and cell cycle progression, and promoted cell apoptosis. miR‐26a targets the DNA methyltransferase 3B transcript and that its inhibition may result in the up‐regulation of MEG3 44. miR‐140‐5p could inhibit the invasion and migration of TSCC cells. LAMC1, HDAC7 and PAX6, clustered into migration‐related genes, were validated to be direct targets of miR‐140‐5p 45. miR‐639 was significantly down‐regulated in TGFbeta‐treated SCC9 cells. Ectopic expression of miR‐639 with miRNA mimics effectively blocked TGFbeta‐induced epithelial–mesenchymal transition (EMT) in SCC9 and CAL27 cells. Clinically, reduced miR‐639 expression was associated with metastasis in TSCC and poor patient survival. FOXC1 was identified as a direct target of miR‐639 46. miR‐219 was significantly down‐regulated in TSCC tissues and cell lines. miR‐219 overexpression remarkably suppressed cell proliferation, colony formation, migration and invasion of TSCC cells. In addition, protein kinase CI (PRKCI) was identified as a target of miR‐219, and overexpression of PRKCI could significantly attenuated the tumour suppressive effects of miR‐219. Furthermore, PRKCI inversely correlated with miR‐219 in TSCC tissues 47. Cao *et al*. reported that miR‐26b expression was decreased in human TSCC and was associated with clinical stage, lymph node metastasis and survival prognosis. Ectopic expression of miR‐26b suppressed the proliferation and metastasis of human TSCC cells. In addition, prostaglandin‐endoperoxide synthase‐2 (encoding COX‐2) was the functional target of miR‐26b 48 (Table 2).

**Table 2:** Functional characterization of the deregulated miRNAs in TSCC

Name	Up‐ or down‐regulation	Target gene	Role	References
miR‐184	Up	c‐Myc	Oncogene	37
miR‐21	Up	TPM1	Oncogene	1
miR‐24	Up	CDKN1B	Oncogene	38
miR‐133a, miR‐133b	Down	PKM2	Tumour suppressor	32
miR‐222	Down	MMP1	Tumour suppressor	33
miR‐7	Down	IGF1R/Akt	Tumour suppressor	39
miR‐138	Down	RhoC and ROCK2	Tumour suppressor	40
miR‐138	Down	CLNS1A, GNAI2, SLC20A1, EIF4EBP1, RhoC	Tumour suppressor	41
miR‐99a	Down	mTOR	Tumour suppressor	3
miR‐195	Down	Cyclin D1, Bcl‐2	Tumour suppressor	42
miR‐25‐3p	Down	cyclinD1, AKT, FOXO1	Tumour suppressor	43
miR‐26a	Down	DNA methyltransferase 3B	Tumour suppressor	44
miR‐140‐5p	Down	LAMC1, HDAC7, PAX6	Tumour suppressor	45
miR‐639	Down	FOXC1	Tumour suppressor	46
miR‐219	Down	PRKCI	Tumour suppressor	47
miR‐26b	Down	PTGS2	Tumour suppressor	48

TPM1: tropomyosin 1; CDKN1B: cyclin‐dependent kinase inhibitor 1B; PKM2: pyruvate kinase type M2; MMP1: matrix metalloproteinase 1; PRKCI: protein kinase CI; IGF1R: insulin‐like growth factor 1 receptor; CLNS1A: chloride channel, nucleotide‐sensitive 1A; GNAI2: G protein alpha inhibiting activity polypeptide 2; RhoC: Rho‐related GTP‐binding protein C; SLC20A1: solute carrier family 20, member 1; EIF4EBP1: eukaryotic translation initiation factor 4E binding protein 1; mTOR: mammalian target of rapamycin; TSCC: tongue squamous cell carcinoma; PTGS2: prostaglandin‐endoperoxide synthase‐2.

## miRNAs contribute to the chemoresistance in tongue squamous cell carcinoma

Resistance to anticancer agents usually leads to failure of chemotherapy and is a challenge for clinicians in management of TSCC. miR‐214 and ‐23a were increased in patients with chemoresistance against cisplatin in the Tca/cisplatin cells while miR‐21 was decreased as with chemosensitivity for cisplatin in the Tca/cisplatin cells. Intervention of these three miRNAs could decrease the chemoresistance against cisplatin in Tca/cisplatin cells. Transfection of anti‐miR‐23a into the Tca/cisplatin cells could increase the topoisomerase II beta (TOP2B) protein expression. These results showed that miR‐21 served as a chemosensitive miRNA, while miR‐214 and ‐23a served as chemoresistant miRNAs in the TSCC lines. miR‐23a is an up‐stream regulator of TOP2B to realize the chemoresistance of cisplatin 34. miR‐200b and miR‐15b were the most significantly down‐regulated miRNAs in CAL27‐res cells. Ectopic expression of miR‐200b and miR‐15b effectively reversed the phenotype of EMT in CAL27‐res and SCC25‐res cells, and sensitized them to chemotherapy. B lymphoma Mo‐MLV insertion region 1 homologue (BMI1) was a target for miR‐200b and miR‐15b. *In vivo*, enforced miR‐200b or miR‐15b expression suppressed metastasis of TSCC xenografts established by CAL27‐res cells. Clinically, reduced miR‐200b or miR‐15b expression was associated with chemotherapeutic resistance in TSCCs and poor patient survival 36. Liu *et al*. found that cisplatin‐induced chemoresistance in TSCC cell lines underwent EMT and was accompanied by enhancing metastatic potential (migration and invasion *in vitro*), miR‐181a down‐regulation and Twist1 up‐regulation. Functional analyses indicated that miR‐181a reversed chemoresistance, inhibited EMT and metastatic potential in TSCC cells. Twist1 was confirmed as a direct miR‐181a target gene by luciferase reporter gene assays 49. The expression of miR‐21 in tumourous tissue was significantly higher compared with adjacent normal tissue and loss of programmed cell death 4 (PDCD4) expression was observed in TSCCs. Transfection of miR‐21 inhibitor induced sensitivity of TSCC cells (Tca8113 and CAL‐27) to cisplatin. Tongue squamous cell carcinoma cells transfected with PDCD4 siRNA became more resistant to cisplatin therapy. The enhanced growth‐inhibitory effect by miR‐21 inhibitor was weakened after the addition of PDCD4 siRNA. Suppression of miR‐21 or PDCD4 could significantly promoted or reduce cisplatin‐induced apoptosis respectively 50 (Table 3).

**Table 3:** miRNAs contribute to the chemoresistance in tongue squamous cell carcinoma

Name	Chemotherapy drugs	Expression	Target gene	Chemoresistance	References
miR‐214	Cisplatin	Up		Chemoresistant	34
miR‐23a	Cisplatin	Up	TOP2B	Chemoresistant	34
miR‐21	Cisplatin	Down		Chemosensitive	34
miR‐200b	Cisplatin	Down	BMI1	Chemosensitive	36
miR‐15b	Cisplatin	Down	BMI1	Chemosensitive	36
miR‐181a	Cisplatin	Down	Twist1	Chemosensitive	49
miR‐21	Cisplatin	Up	PDCD4	Chemoresistant	50

BMI1: B lymphoma Mo‐MLV insertion region 1 homologue; PDCD4: programmed cell death 4.

## Concluding remarks and future perspectives

Actuating evidence has suggested the importance of miRNA deregulations in the development of human malignancies, which has shed new light on novel therapeutic, diagnostic and prognostic strategies for various cancers, including TSCC 28, 42, 51, 52, 53. MicroRNA microarray profiling has pinpointed some significant deregulated miRNAs that could hold prognostic values for TSCC 1. Moreover, the functional roles of specific miRNAs as tumour suppressors or oncogenes render them attractive targets for therapeutic intervention 42, 54, 55. However, scientists and clinicians are still facing many difficulties in miRNA study. Specifically, miRNA‐based therapy is not currently used in clinic patients because of miRNA expression could be tissue‐specific and influenced by many factors, such as infection, hypoxia, pathology and drug treatment 34, 56, 57, 58, 59. Moreover, a single miRNA could regulate the expression of hundreds of genes including oncogenes and tumour suppressor genes and could have opposite actions in different cancer types 60, 61, 62. Nevertheless, with a more comprehensive understanding of miRNA deregulation in TSCC, it is hopeful that miRNAs will achieve clinical utility at last.

## Conflicts of interest

The authors declare no conflict of interest.
